# Adipose-Derived Mesenchymal Stem Cells: Are They a Good Therapeutic Strategy for Osteoarthritis?

**DOI:** 10.3390/ijms19071926

**Published:** 2018-06-30

**Authors:** Elena Damia, Deborah Chicharro, Sergio Lopez, Belen Cuervo, Monica Rubio, Joaquin J. Sopena, Jose Manuel Vilar, Jose Maria Carrillo

**Affiliations:** 1 Bioregenerative Medicine and Applied Surgery Research Group, Department of Animal Medicine and Surgery, CEU Cardenal Herrera University, CEU Universities, C/Tirant lo Blanc, 7, Alfara del Patriarca, 46115 Valencia, Spain; elena.damia@uchceu.es (E.D.); debora.chicharro@uchceu.es (D.C.); belen.cuervo@uchceu.es (B.C.); mrubio@uchceu.es (M.R.); jsopena@uchceu.es (J.J.S.); jcarrill@uchceu.es (J.M.C.); 2 Garcia Cugat Foundation CEU UCH Chair of Medicine and Regenerative Surgery, 08006 Barcelona, Spain; 3 Department of Animal Pathology. Instituto Universitario de Investigaciones Biomédicas y Sanitarias. University of Las Palmas de Gran Canaria, 35416 Las Palmas de Gran Canaria, Spain; sergiolopezbarbeta@gmail.com

**Keywords:** osteoarthritis, mesenchymal stem cells, regenerative medicine

## Abstract

Osteoarthritis (OA) is a major cause of disability in elderly population around the world. More than one-third of people over 65 years old shows either clinical or radiological evidence of OA. There is no effective treatment for this degenerative disease, due to the limited capacity for spontaneous cartilage regeneration. Regarding the use of regenerative therapies, it has been reported that one option to restore degenerated cartilage are adipose-derived mesenchymal stem cells (ASCs). The purpose of this review is to describe and compare the efficacy of ASCs versus other therapies in OA. Methods: Recent studies have shown that ASCs exert paracrine effects protecting against degenerative changes in chondrocytes. According to the above, we have carried out a review of the literature using a combination of osteoarthritis, stem cells, and regenerative therapies as keywords. Results: Conventional pharmacological therapies for OA treatment are considered before the surgical option, however, they do not stop the progression of the disease. Moreover, total joint replacement is not recommended for patients under 55 years, and high tibia osteotomy (HTO) is a viable solution to address lower limb malalignment with concomitant OA, but some complications have been described. In recent years, the use of mesenchymal stem cells (MSCs) as a treatment strategy for OA is increasing considerably, thanks to their capacity to improve symptoms together with joint functionality and, therefore, the patients’ quality of life. Conclusions: ASC therapy has a positive effect on patients with OA, although there is limited evidence and little long-term follow-up.

## 1. Introduction

Osteoarthritis (OA) is a progressive degenerative disease of the joint characterized by gradual degradation of hyaline articular cartilage and sclerosis of bone. This cartilage is composed by type II collagen and proteoglycans. An alteration in the replacement of the proteoglycan and type II collagen network leads to the loss of function of the cartilage [1]. This disease, worldwide, is considered to be the fourth leading cause of disability [2] and the second cause of inability to work in men [3]. OA is the most common articular disease in adults, and knee OA is the most common location. Although, OA also affects other large-weight-bearing joints, such as hip, hands, feet, and spine [4]. Hip and knee OA are leading causes of disability worldwide [5]. The disease is characterized, at first, by a molecular derangement (alteration of joint tissue metabolism) followed by physiologic/anatomic damages (cartilage degradation, bone remodeling, osteophyte formation, joint inflammation), culminating in a loss of normal joint function [6].

In the United States, 27 millions of people suffer from clinical OA, and the treatment costs 185.5 billion dollars per year [7]. On the other hand, this pathology is the fourth leading cause of disability in Asia [2]. In addition, this chronic degenerative disease of articular cartilage has a current prevalence of 12% in the population over 60 years old, which will increase in the next 20 years [8,9]. It has also been reported that its incidence has doubled in women, and tripled in men, in recent years [10].

Several agents have been associated with a higher risk of suffering OA, such as genetic predisposition, obesity, previous trauma, and age. It has been demonstrated that the risk of developing post-traumatic OA increases by up to four times in people over the age of 50 [11]. As one ages, the chondrocytes, that contribute to 5% of the volume of the articular cartilage, decrease their regenerative response, leading to progressive loss of the articular surface resulting in cartilage degeneration with loss of matrix (which confers the biomechanical properties to the articular cartilage and constitutes the 95% of the tissue), which can result in a complete loss of joint surface. Moreover, chondrocytes produce mediators of inflammation (cytokines, chemokines, and proteolytic enzymes) that induce serious damage [12].

Pain is one of the first symptoms, leading to movement disability and impaired quality of life [9,13]. Synovial inflammation, cartilage breakdown, and bone remodeling are associated with OA chronic pain, and the mechanism responsible for pain involves structural changes and alterations in peripheral transduction and central processing of painful sensory inputs [14]. Consequently, ideal treatment should obtain analgesia, stopping progression of chondral degeneration; modify cartilage structure and revert damage; and finally, improve joint function [15].

While conventional therapies, such as physical therapy, glucosamine and chondroitin sulfate supplementation, arthroscopic surgery, or biological therapies, such as chondrocyte implantation, have little significant results, regenerative medicine (RM), has been demonstrated to be a great option in articular cartilage regeneration [16]. On the other hand, various surgical procedures have been performed to regenerate articular cartilage but have achieved limited success, including abrasion arthroplasty, subchondral drilling, and microfracture [17]. Mesenchymal stem cells (MSCs) are considered to be a promising candidate for cartilage regeneration, due to their ability to differentiate towards cartilage and bone cells and secrete trophic factors with regenerative functions [18]. The paracrine effect and anti-apoptotic, anti-inflammatory and anti-aging functions of these stem cells, is fundamental for the regeneration process. Recently, an anti-aging effect of the conditioned medium of adipose-derived mesenchymal stem cells (ASCs) on OA chondrocytes has been reported, featured by downregulation of senescence markers induced by inflammatory stress [19]. Stem cells promote biological processes, such as vascularization, cell proliferation, differentiation, and modulation of the inflammatory process [20], and can be isolated, among others, from bone marrow, adipose tissue, umbilical cord blood, and placenta [21,22]. It is currently admitted that there are MSCs within the connective tissue of virtually all organs [20]. In humans, ASCs showed a greater capacity for proliferation than the rest of the human MSCs [23], moreover, these cells maintain the differentiation potential after a longer time of culture [24], and the age of donors has less effect on the proliferation of them; this is important in elderly patients with osteoporosis [25].

ASCs were first identified in the early 2000s, and demonstrated to have self-renewal ability and multilineage differentiation potential [26]. These cells have a several benefits: faster and easier expansion in culture, more passage cells that retain stem cell phenotypes, pluripotency [27], less susceptibility to age, and less morbidity of patients [28], furthermore, compared with bone marrow-derived mesenchymal stem cells (BMMSCs), ASCs do have an equal potential to differentiate into cells and tissues of mesodermal origin, such as adipocytes, cartilage, bone, and skeletal muscle. On the other hand, the easy and repeatable access to subcutaneous adipose tissue and the simple isolation procedures provide a clear advantage [29].

To establish the efficacy of treatment with this regenerative therapy and assess, in the case of OA, the quality and thickness of the cartilage, long-term patient controls are needed [30]. Different studies have shown that the application of MSCs as therapy in the treatment of OA has improved the symptoms suffered by patients, particularly after more than 6 months of follow-up [10,30,31].

Recent studies have focused on BMMSCs for chondrogenesis, but the clinical use of these cells has presented disadvantages, such as, donor site morbidity, pain and low cell number upon harvest [32]. On the other hand, ASCs are a positive alternative treatment for OA treatment as in vitro studies have proven they contain CD73, CD90, CD105, and CD106 markers, which are necessary for cell differentiation into cartilage, and moreover, in vivo studies have also reported good results [33]. For all of the abovementioned reasons, the aim of the present study is to review the application of MSCs in OA, with particular emphasis on the use of ASCs versus other therapies.

## 2. Results

The prevalence and incidence of OA have increased globally, but pharmaceutical or surgical therapies have limited efficacy in halting OA progression. Among conservative therapies, nonpharmacological treatment (physiotherapy, weight management), systemic pharmacological treatment (analgesics, nonsteroidal anti-inflammatory drugs, glucosamine, and chondroitin sulfate) and injections of intra-articular (IA) therapies are described. Local delivery of corticoids and hyaluronic acid (HA) are approved treatments by United States Food and Drug Administration (US FDA)/European Medicines Agency (EMA), however, some adverse effects have been described, such as inflammation or pain and septic arthritis at the site of injection [34].

When OA advances, some surgical treatments can rebuild the degenerated cartilage, but they do not stop the articular inflammatory process established. These treatments are arthroscopic debridement, microfracture/osteoplasty, and chondrocyte implantation techniques, such as autologous chondrocyte implantation and matrix-induced autologous chondrocyte implantation [35]. The latter is the only cell therapy approved by the FDA [36].The application of such interventions remains limited, due to the necessity of additional surgery for harvesting the donor autograft cartilage, and the poor integration of the grafted defect with the surrounded cartilage [37].

Due to the limitations regarding OA conventional therapies, for example, the pharmacological therapy could produce serious gastrointestinal, renal, and cardiac adverse effects, and some of them can be a threat to life or they can leave a permanent disability. On the other hand, the surgical option, such as microfracture, has been used for the last 20 years, but hyaline cartilage has a limited capacity for regeneration. In recent years, the interest for new therapies, such as IA injection, autologous blood therapies, and MSCs is increasing considerably. Autologous conditioned serum (ACS) is a cell-free treatment obtained by incubating venous blood in a specialized syringe, where blood cells release anti-inflammatory cytokines and growth factors (GFs), such as transforming growth factor-β (TGF-β) [38]. It has been demonstrate that ACS therapy is more effective than HA injection [39] and the treatment with ACS and physiotherapy reduce chronic pain in knee OA [40]. Plasma rich in growth factors (PRGFs), is a type of platelet rich plasma (PRP) preparation. It is an autologous product with a moderate concentration of platelets, multitude of GFs, and absence of leukocytes. It provides an anabolic effect on the resident cells, and due to its potential to inhibit inflammation, relieves OA symptoms [41]. When PRGF is injected IA, it provides a three-dimensional network in the joint composed of fibrin that contains binding sites for cell adhesion, as well as proteins that form the microenvironment leading to different adhesion molecules and cells that help biological cartilage repair [42]. Recently, it has been reported that PRGF injection is an effective option to decrease pain and improve function in patients with symptomatic mild to moderate knee OA, in the 6 month follow-up [43]. In recent years, the release of ASCs with PRP has been reported to improve the proliferation and chondrogenesis of this type of stem cells [44,45], suggesting new applications in RM for the management of osteochondral defects.

Currently, RM, that aims to promote regenerative or reparative phenomena over the degenerative processes, is in full swing. Among these therapies are the already mentioned PRGF and the MSCs.

### 2.1. Mesenchymal Stem Cells

MSCs are defined as those cells that meet the criteria established by the International Society of Cellular Therapy. These criteria include an ability to adhere to plastic, the expression of a number of cell markers, including CD105, CD73, and CD90 while undergoing multilineage differentiation, and the ability to self-renew [46]. Those multipotent adult stem cells synthesize mediators (cytokines, neuroregulatory peptides, trophic factors) which participate in tissue repair and regulate inflammatory and immune responses [47]. These adult stem cells could be induced to differentiate exclusively into the adipocytic, chondrocytic, or osteocytic lineages. It has been identified that individual stem cells, when expanded to colonies, retained their multilineage potential [48].

MSCs can be obtained from various adult tissues, for example, bone marrow, umbilical cord, skeletal muscle, synovial capsule, and adipose tissue. This last origin, has a number of advantages over the others, because adipose tissue is abundant and easy to obtain, and can be obtained in large amounts, using local anesthesia and causing minimal discomfort [49]. Subcutaneous fat tissue is the most accessible source, but in recent studies, other sources for obtaining autologous ASCs as the supra- and infrapatellar fat pads have been described [50]. Bone marrow aspirate has a paucity of MSCs, comprising 0.001–0.02% of the mononucleated cell population, in comparison to ~1–7% of the mononucleated cell population within adipose tissue [51,52]. Moreover, adipose tissue is considered a primary source, because it contains 500 times more MSCs than the same volume of bone marrow [53]. Furthermore, bone marrow harvested from the iliac crest is painful, and increases the risk of infection [54]. Recently, some reports demonstrate that IA injection of allogenic ASCs combined with HA could stop OA progression and promote cartilage regeneration [55]. Additionally, a successful management of a post-traumatic chondral defect using IA autologous ASC therapy has been suggested [35].

The FDA regulates the use of adult stem cells. This agency adopted 21 CFR 1271, which modified its jurisdiction over human cells and tissues to include any “transfer into a human recipient”. Previously, the code was specified transfer “into another human,” excluding autologous cells. Since then, cells that are more than “minimally manipulated,” even if they are intended for autologous use, and are subject to similar regulations as manufactured drugs [56]. More research studies on the use of MSCs in OA treatment would allow the FDA and physicians to provide patients with a more confidently alternative, minimally invasive treatment options that may significantly slow disease progression.

#### 2.1.2. The Role of Mesenchymal Stem Cells in Osteoarthritis

The mechanism by which MSCs cause cartilage regeneration is not clear. It has been postulated that these cells may act on subchondral bone, forming the primary repair cartilage [57]. The injection of MSCs in joint cavity is a novel therapy that improves OA symptoms due to their ability to stimulate local repair and regeneration of damaged joint tissues, and to reduce inflammation and associated pain [16]. MSCs modulate the inflammatory response by causing the suppression of inflammatory T-cell proliferation and inhibition of monocyte and myeloid dendritic cell maturation [58]. Moreover, the anti-inflammatory capacity can be stimulated by various pro-inflammatory cytokines (IL-6, tumor necrosis factor and interferon gamma) [59]. Furthermore, these stem cells secrete reparative cytokines, including TGF-β, vascular endothelial growth factor (VEGF), and epidermal growth factor (EGF), which are responsible for a trophic effect that produces the local tissue repair [60]. Additional in vitro studies have demonstrated that GFs, such as TGF-β and insulin-like growth factor 1 (IGF-1), can stimulate MSCs towards chondrocytes. These chondrocytes, derived from MSCs, have the same expression of type II collagen that mature adult chondrocytes, moreover, this type II collagen provides tensile strength in the joint [61].

It has been demonstrated that after IA injection of MSCs (Table 1), these cells were found in the synovial membrane and they expressed molecules with anti-inflammatory and chondrogenic properties. MSCs could help to establish a regenerative microenvironment at the site of release, which would improve the recruitment, activation, and differentiation of endogenous stem cells with the potential to repair the articular cartilage [62].

With the aim of achieving a prolonged regenerative activity in the OA joint, several studies propose using MSCs activated with biomolecules to potentially improve chondral and osteochondral lesion repair. The need to use bioactive scaffolds was proven by the fact that in the knee joint of experimental mice, only 15% of ASCs injected were detectable at 1 month post-injection, and this number decreased to 1.5% in 6 months [63]. This type of MSC co-delivery with scaffolds could have better retention, aggregation, and viability of these cells; moreover, the proliferation, migration, and chondrogenic differentiation improve in scaffolds with large pore size [64].

Some in vitro studies have demonstrated the beneficial effects of scaffolds (CDM, poly l-glutamic acid/chitosan, TGF-β1-conjugated chitosan hydrogel), such as regeneration of hyaline cartilage, enhancing ASC chondrogenesis and reparation of full-thickness cartilage defects [65,66,67]. More scaffolds include fibrin, gelatin and collagen (protein-based scaffolds), and alginate or agarose, among others (carbohydrate-based scaffolds). Other scaffolds that maintain a three-dimensional structure include the hydrogel family and hydrophilic polymer [36].

#### 2.1.3. Mesenchymal Stem Cells Exosomes in Osteoarthritis

Exosomes are a type of secreted membrane vesicles produced by different cells. Some types of exosomes have been shown to confer immunosuppressive effects in different disease models, among others, rheumatoid arthritis [77]. MSC exosomes are accepted as the principal therapeutic agents present in MSC secretion, and are adequate to mediate the many reported therapeutic options of MSCs [78]. Zhang et al., (2016) have reported that human MSC exosomes promote cartilage regeneration in an immunocompetent rat osteochondral defect model, so MSC exosomes help to regenerate the damaged articular cartilage in OA. The mechanism of action of MSC exosomes in that study were accelerated neotissue filling and enhanced matrix synthesis of type II collagen and sulphated glycosaminoglycan [79].

Another study demonstrates that weekly IA injections of human embryonic MSC exosomes induced a regeneration cartilage and subchondral bone over a period of 12 weeks in an adult immunocompetent rat model [79]. Definitely, the efficacy of MSC-based therapies has been assigned to the paracrine secretion of trophic factors, and exosomes have a fundamental role in mediating tissue repair, thus, exosomes represent a novel therapeutic option for OA.

## 3. Discussion

ASCs have a series of advantages over other types of cells, because adipose tissue is abundant and easy to obtain. On the other hand, these cells have a high in vitro proliferation capacity and fibroblastic morphology, and they can adhere well to the culture plate. Furthermore, they have a low risk of rejection [80]. ASCs can be isolated from the stromal vascular fraction (SVF) of adipose tissue. The cells are obtained by liposuction, followed by collagenase digestion, centrifugation, and dilution. SVF includes ASCs, as well as other cells, including pericytes, vascular adventitia cells, fibroblasts, preadipocytes, monocytes, macrophages, and red blood cells. The SVF product has 500,000 to 2,000,000 cells per gram, of which 1 to 10% are considered ASCs [81].

It has been calculated that there are approximately 1 × 10^5−6^ ASCs in 1 ml of lipoaspirate, while there are 50–675 BMMSCs in 1 mL of bone marrow aspirate [82]. Another alternative for OA treatment with MSCs are human umbilical cord-derived MSCs (hUC-MSCs). These cells enhance the proliferation of OA chondrocytes and downregulate the expression of inflammatory cytokines, moreover, the co-culture of hUC-MSCs and OA chondrocytes may to be a therapeutic option for OA [22]. hUC-MSCs could be an alternative to BMMSCs for clinical applications, due to their easy preparation and low risk of viral contamination. They can differentiate into the three germ layers that promote tissue and organ repair and modulate immune responses [83]. In this review, we have reported the different types of treatment that are used conventionally in joint degenerative disease. Our discussion focuses on comparing ASCs with others stem cells most commonly used in the OA treatment in recent years.

### 3.1. Adipose-Derived Mesenchymal Stem Cells

In human medicine, it has been shown that MSCs are a clinical promise for articular cartilage regeneration. Several authors reported studies that demonstrate the effectiveness of MSCs in OA treatment. In relation to ASCs, the first case report was published in 2001 [21]. During the last decade, these cells have attracted great interest because they have been demonstrated to be safe and efficient for articular cartilage regeneration in several trials. In recent years, IA injection of ASCs in knee OA showed clinical, radiological, arthroscopic, and histological evidence at 6-month follow-up [74]. Among other studies, the IA injection of these stem cells (isolated from abdominal subcutaneous fat tissue) in severe knee OA, reported that clinical outcomes (pain, function knee, return to sport) of the low- and medium-dose groups tended to deteriorate after 1 year, while those of the high-dose group tended to plateau after 1 year, until 2 years [10]. Recently, Spasovski et al. (2018) have demonstrated that the use of ASCs from subcutaneous fat in knee OA improves clinical symptoms and reduces pain at 3 months, obtaining the best results at 6 months [30]. ASC therapy in OA has shown chondrogenesis potential, both for the infrapatellar- and suprapatellar-derived ASCs [50,84]. A greater chondrogenesis potential has been reported by infrapatellar ASCs compared to suprapatellar in vitro and in vivo [84,85]. In addition, the suprapatellar-derived ASCs transplantation in a severe knee OA mouse model diminished inflammation and cartilage degenerative grade, increasing the synthesis of glycosaminoglycan and inducing endogenous chondrogenesis [50]. These effects may be due to ASCs-mediated reduction of pro-inflammatory cytokines and chemokines, apoptosis of chondrocytes, hypertrophic and fibrotic chondrocyte phenotypes, and collagenases [86].

One of the limitations of the studies that describe the use of ASCs in OA is the short follow-up period, Joe at al. (2014) and Pers et al. (2016) reported the efficacy of IA injections of these cells for the treatment of knee OA, but their follow-up period was only 24 weeks [72,74]. However, Song et al. (2018) have reported the first study that has demonstrated the efficacy of ASCs therapy in knee OA with long-term follow-up of 96 weeks with repeated injections. These patients showed improvement in pain, function, and cartilage volume of the knee joint with repeated IA injections of these cells [87].

### 3.2. Bone Marrow Mesenchymal Stem Cells

Pittenger et al. (1999) isolated MSCs from bone marrow adult cells and since then they have been used to treat chondral defects [48]. Some studies in patients with OA treated with BMMSCs obtained good results, reducing the symptoms and, therefore, increasing patient satisfaction. In a study carried out in 24 patients with knee OA infiltrated with BMMSCs, histological and arthroscopic improvement was observed [88]. Another report by Kuroda et al. (2002) concluded that the transplantation of autologous BMMSCs promotes the repair of large defects of focal articular cartilage in young and active patients [89]. Recently, safety of IA injection of BMMSCs was confirmed in 12 OA patients. They showed pain relief and improvement of cartilage quality at 2 years post-treatment [90]. Regarding the regulation of the inflammatory process in OA, Zhang et al. (2016) reported that the co-cultivation of BMMSCs with chondrocytes from patients with OA increases cell proliferation of chondrocytes and inhibits inflammatory activity in OA [91]. In a study of knee OA treatment in 13 patients with in vitro expanded BMMSCs at 12 months, a significant improvement in the thickness of knee cartilage in the femoral and tibial plates was shown [73]. Good results have also been reported with the application of BMMSCs in large [92] and small joints [71] with microfracture.

### 3.3. Human Umbilical Cord-Derived Mesenchymal Stem Cells

Some studies have demonstrated that chondrocytes secrete the same cytokines and induce human stem cells to differentiate into chondrocytes [93,94]. It has been reported that the hUC-MSCs improve the viability of OA-degenerated chondrocytes. A study carried out in Shangai suggested that the secretion of hUC-MSCs enhanced chondrocyte proliferation and showed that these stem cells increased expression of chondrogenic genes (*aggrecan, sox-9, collagen II*), indicating chondrocytes promoted chondrogenic differentiation of hUC-MSCs, compared to the control group. They also postulated that hUC-MSCs inhibited inflammatory activity in OA chondrocytes [22]. In the same line, Zheng et al. (2013) reported the chondrogenic differentiation of hUC-MSCs by co-culture with rabbit chondrocytes [95]. In other studies, hUC-MSCs were reported to inhibit the expression of some inflammatory factors [96,97]. Definitely, hUC-MSCs could regulate inflammatory activity and the proliferation of chondrocytes in OA. 

Therapeutic options of OA depend on each individual case and multiple factors, such as the disease progression, degeneration degree of articular cartilage, affected joint, and patient’s expectations. The gold standard in the treatment of OA is total joint replacement, but surgery is not suitable for patients under 55 years [98], however, HTO is intended to transfer the mechanical axis from medial to slightly lateral, to the midline of the knee, to decrease the load and subsequently delay OA [99] in 40–60 years old patients [100]. Some studies showed that the regenerative process began after realignment [99], but different complications have been described, such as aseptic nonunion and deep infection [101]. MSCs may be a safe and alternative treatment strategy for OA, due to that this therapy has different advantages: it is applied in all joints, the injections are repeatable, and it is minimally invasive [12].

## 4. Materials and Methods

The authors searched PubMed English languages articles using a combination of “osteoarthritis”, “stem cells”, “regenerative therapies”, and “adipose-derived mesenchymal stem cells” as keywords. After the first selection of the main articles based on OA and conventional treatments, studies of OA and stem cells were selected, and there were a total of about 900 articles in the last decade. Special attention has been drawn to original analyses and studies, around 300 studies, of which we chose about 80 articles dedicated to ASCs and were published in the last 10 years. Other searches were executed using bibliographies of articles found in the primary and secondary search. One limitation in this review, is the fact that our methods, while rigorous, did not follow any formal guidelines for a systematic review (e.g., the Preferred Reporting Items for Systematic Reviews and Meta-Analyses (PRISMA) guidelines).

## 5. Conclusions

OA is a major health problem, especially in the elderly population. Cellular therapy is an emerging modality for the treatment of OA, even the combination of conventional treatments with the application of MSCs is a therapeutic option to improve the quality of life of patients with OA. In recent years, the interest in MSCs as a therapeutic option in OA is due to the facility of harvesting, preparation, and implantation without surgery, the capacity to stimulate local repair and regeneration of damaged joint tissues, and the ability to reduce inflammation and associated pain. ASCs have different advantages, including easy cryopreservation, faster expansion in culture, more passage cells that retain stem cell phenotypes and pluripotency, and less susceptibility to aging together with lower morbidity of patients. Moreover, the obtention of adipose tissue is much less expensive than bone marrow, with less invasive intervention and available in greater quantities.

ASCs in OA may offer an exciting possibility to improve function, pain, and cartilage volume of the joint, suggestive of a good therapeutic strategy for OA. Despite this, further long-term studies are needed to prove and evaluate the effectiveness of ASCs in OA treatment and their safety capacity. However, it is important to establish a standardized therapeutic protocol for this biological therapy, and assess each patient and each pathology individually.

## Figures and Tables

**Table 1:** Research on osteoarthritis with MSC-based therapy.

Study	Model	MSCs Type	OA Location	Results
Garay-Mendoza et al., 2018 [15]	Human	BMMSCs	Knee	Improvement in knee pain and quality of life since first evaluation until the last one at 6 months
Sun et al., 2018 [68]	Rabbit	ASCs + TGF-β3 poly-lactic-co-glycolic acid Microspheres	Knee	Promoted cartilage regeneration and lessened the severity of OA in vivo
Desancé et al., 2018 [69]	Equine	UCBMSCs	In vitro	High proliferative capacity and differentiated into osteoblasts and chondrocytes. Have a great potential for cartilage tissue engineering
Freitag et al., 2017 [35]	Human	Arthroscopy with removal of a chondral loose body + ASCs	Post-traumatic chondral defect of the patella	Complete regeneration of hyaline-like cartilage within the defect and improvement of the pain and function
Abbas 2017 [70]	Human	BMMSCs + cartilage fragments	Osteochondral bone samples from patients with total knee arthroplasty and a central drill defect (human ex vivo osteochondral defect model)	Improvement in chondrogenic differentiation and positive staining for type II collagen antibodies
Murphy et al., 2017 [71]	Human	BMMSCs	First Carpometacarpal joint	Functional and symptomatic relief for the patients
Pers et al., 2016 [72]	Human	ASCs	Knee	Patients treated with ASCs experienced significant improvements in pain levels and function knee compared with baseline.
Rich et al., 2015 [73]	Human	BMMSCs	Knee	Significantly improved the knee injury and Osteoarthritis Outcome Score and knee cartilage thickness (measured by magnetic resonance imaging), indicating that they may enhance the functional outcome as well as the structural component
Jo et al., 2014 [74]	Human	ASCs	Knee	Improve function and pain of the knee joint without causing adverse events, and reduce cartilage defects by regeneration of hyaline-like articular cartilage
Wu et al., 2014 [75]	Rat	SMSCs + fibrin/chitosan scaffold + TGF-β3	Temporomandibular Joint	Fibrocartilage formation with deposition of Col1 and Col2
Chen et al., 2013 [76]	Rabbit	BMMSCs	Temporomandibular Joint	Enhance the regenerative process of cartilage repair at the early stage of Temporomandibular joint OA

MSCs mesenchymal stem cells, BMMSCs bone marrow mesenchymal stem cells, OA osteoarthritis, TGF-β3 transforming growth factor β3, UCBMSCs umbilical cord blood mesenchymal stem cells, ASCs adipose-derived stromal cells, SMSCs synovial mesenchymal stem cells, Col1 and Col2 type 1 and type 2 collagen.

## Abbreviations

ACS	Autologous conditioned serum
ASCs	Adipose-derived mesenchymal stem cells
EGF	Epidermal growth factor
EMA	European Medicines Agency
FDA	Food and Drug Administration
GFs	Growth factors
HA	Hyaluronic acid
hUC-MSCs	Human umbilical cord mesenchymal stem cells
IA	Intra-articular
IGF-I	Insulin-like growth factor-I
MSCs	Mesenchymal stem cells
OA	Osteoarthritis
PRGFs	Plasma rich in growth factors
PRP	Platelet rich plasma
RM	Regenerative medicine
TGF-β	Transforming growth factor-beta
VEGF	Vascular endothelial growth factor
HTO	High tibia osteotomy
